# Supplementary material for: Polymorphisms in Pfkelch13 domains before and after the introduction of artemisinin-based combination therapy in Southwest Nigeria
Source: PLoS One. 2025 Mar 31;20(3):e0316479. doi: 10.1371/journal.pone.0316479 (PMC11957316; doi:10.1371/journal.pone.0316479)
Supplement: Supporting information 2 — (ZIP) [file pone.0316479.s002.zip › 132KN2F_PREMIX_Plate_KELCH2_B12.pdf]

Samples: 12074
Bases: 794
Average spacing: 16

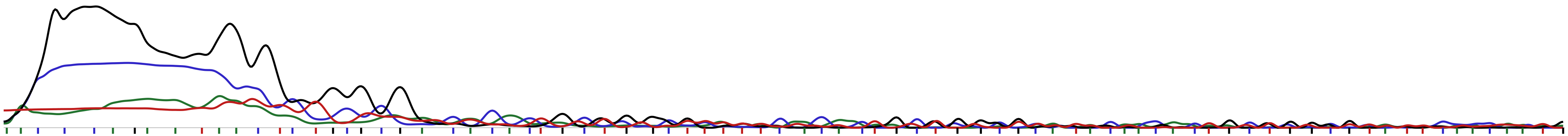

10 20 30 40 50 60 70 80
A A C C C A G A A T A A C T C T G C G C G A C A C A C T G C T G G C T T T T T C A C A C T G C T G G C G C A T A C A A C A C T G C T G G C G T A T T C A A C A A T G

90 100 110 120 130 140 150 160
C T G G C G T A T A C A A C A A T G C T G G C G T G T A C A A C A C T G C T G G G G T A G A C A A C A A T G C T G G C G T A G A C A C C A C T G C G G G G T A A T C

170 180 190 200 210 220 230 240 250
A A C A A T G A A C A T G T A A C A A T A T C T C T A A A G A A A A T A T A G G A A A T A A A T A T T T A A A T A A A T T G T T A A A T A A A A A A A A A G A T A C T A T

260 270 280 290 300 310 320 330
T A C A A A T G A A A A T A A T A A T A T T A A T C A T A A T A A T A A T A A T A A T A A T A T G A A T C T G A C T G C A A A T A A T A T A A C T A A T A A T C T T A T

340 350 360 370 380 390 400 410
T A A T A A T A A T A T G A A T T C T C C A A C A A T T A T G A A T A C C A A C A A A A A A G A A A A T T T T T T A G A T G C A G C A A A T C T T A T T A A T G A T G A

420 430 440 450 460 470 480 490 500
T T C T G G A T T A A A C A A T T T A T T T T A A T T T T C A A C T G T G A A A A A T G T A A A T G A T A C T T A T G A A A A G A A A A T T A T T G A A A C G G A A T

Samples: 12074
Bases: 794
Average spacing: 16

510 520 530 540 550 560 570 580
TAAGTGATGCTAATGATTTTGACAATATGGTATGTGATTTCATAATTACTTTTATTAATTGGGAAAAAAAGACACCTATGAAT

590 600 610 620 630 640 650 660
TTTATTCGAGAATAAAAATAATTATTTAAAGATACTAAAGAACTACAAATGGAAAGAGTACCATAGTACAAAAAATTAGAAAAC

670 680 690 700 710 720 730 740
CCCAAAAATATTGTAGAACATAATTACATGATGAAAGAAAGAAATTGATATTGATCTATCTCATGGTACAACACATCAA

750 760 770 780 790
AAAGACATAGTCCACTTGGATTCGATTTGATTAAGAAGATTTACGA

Samples:	12074
Bases:	794
Average spacing:	16
